# Measurement Training and Feedback System for Implementation of family-based services for adolescent substance use: protocol for a cluster randomized trial of two implementation strategies

**DOI:** 10.1186/s13012-019-0874-6

**Published:** 2019-03-11

**Authors:** Aaron Hogue, Sarah Dauber, Molly Bobek, Amanda Jensen-Doss, Craig E. Henderson

**Affiliations:** 1 Center on Addiction, New York, NY USA; 2 Department of Psychology, University of Miami, Miami, FL USA; 3 Department of Psychology, Sam Houston State University, Huntsville, TX USA

**Keywords:** Adolescent substance use treatment, Family-based services, Measurement feedback system, Quality improvement, Facilitation, Cluster randomized implementation trial

## Abstract

**Background:**

This article describes a study protocol for testing the Measurement Training and Feedback System for Implementation (MTFS-I) and comparing two implementation strategies for MTFS-I delivery. MTFS-I is a web-based treatment quality improvement system designed to increase the delivery of evidence-based interventions for behavioral health problems in routine care settings. This version of MTFS-I focuses on family-based services (FBS) for adolescent substance use. FBS, comprising both family participation in treatment and family therapy technique use, have achieved the strongest evidence base for adolescent substance use and are a prime candidate for upgrading treatment quality in outpatient care. For FBS to fulfill their potential for widespread dissemination, FBS implementation must be bolstered by effective quality procedures that support sustainable delivery in usual care.

**Methods/design:**

Adapted from measurement feedback systems for client outcomes, MTFS-I contains three synergistic components: (a) weekly *reporter training modules* to instruct therapists in reliable post-session self-reporting on FBS utilization; (b) weekly *mock session videos* of FBS interventions (5–8 min) for supportive training in, and practice coding of, high-quality FBS; and (c) monthly feedback reports to therapists and supervisors displaying aggregated data on therapist-reported FBS use. MTFS-I is hosted online and requires approximately 20 min per week to complete. The study will experimentally compare two well-established implementation strategies designed to foster ongoing MTFS-I usage: Core Training, consisting of two 3-h training sessions focused on FBS site mapping, selecting FBS improvement goals, and sustaining MTFS-I, followed by routine remote technical assistance; and Core + Facilitation, which boosts Core Training sessions with collaborative phone-based clinical consultation and on-site facilitation meetings for 1 year to promote FBS goal achievement. The study design is a cluster randomized trial testing Core Training versus Core + Facilitation in ten substance use treatment clinics. Study aims will compare conditions on MTFS-I uptake, FBS delivery (based on therapist-report and observational data), and 1-year client outcomes.

**Discussion:**

Study contributions to implementation science and considerations of MTFS-I sustainability are discussed.

**Trial registration:**

ClinicalTrials.gov NCT03342872. Registered 10 November 2017

## Background

### Increasing the effectiveness and sustainability of family-based services for adolescent substance use via quality improvement systems

Discovering and disseminating effective methods to improve the quality of treatment services for adolescent substance use (ASU) within the national behavioral healthcare system is a public health priority [1, 2]. Broadly speaking, ASU treatment quality is considered mediocre to inadequate due to a host of factors headlined by the absence or modest scope of evidence-based services, along with inadequate provider training, lack of reliable quality metrics and quality monitoring procedures, and ineffective system-level policies for supporting quality mandates [3, 4]. To correct these quality deficits, ASU treatment delivery must be supported by effective quality assurance procedures designed to ensure that efficacious treatments are delivered with fidelity, that is, to the intended population, by appropriately trained providers, and in accord with specified principles and procedures [6]. This includes the need for quality metrics [5] that can reliably measure implementation fidelity in routine settings.

A valuable strategy that can buttress quality delivery of evidence-based interventions (EBIs) is use of “learning” quality improvement systems that promote data-driven decision-making [7]. In the behavioral treatment arena, learning systems of this kind consist of treatment quality procedures in which EBI implementation activities are carried out incrementally, implementation and sustainability data are regularly collected and reviewed, and continuous EBI modifications are made to increase fit and/or feasibility (see [8]). Learning systems are intended to ingrain data-driven decision-making into the procedural routines of agencies.

One highly promising means to upgrade the quality of the ASU treatment system is increasing the implementation of family-based services. “Family-based” refers to both *family participation* in services, meaning family member involvement in treatment-related activities; and *family therapy techniques*, the specific interventions that clinicians use to target family members and family processes for change [9]. Family-based services (FBS) have by far the strongest research base for treating ASU, posting an exemplary record of success in comparison to alternative EBIs as well as usual care, and FBS produce the largest average effect sizes by a wide margin [10–12]. FBS also have the strongest empirical support for treating adolescent disruptive behavior (i.e., aggression, conduct disorder, delinquency) [10, 11], and many FBS studies report significant reductions in anxiety and depression symptoms as well as gains in prosocial functioning [9–11]. FBS are strongly endorsed by federal agencies, national service provider organizations, policy-making groups, and clinicians who treat youth in routine behavioral care [13, 14].

The primary implication of these scientific and policy mandates for improving treatment quality is clear: To promote effective implementation of FBS for ASU, pragmatic quality procedures for sustaining high-fidelity FBS need to be developed. This article describes an effectiveness-implementation hybrid study protocol designed to achieve this goal. The protocol is a cluster randomized trial that will test a Measurement Training and Feedback System for Implementation (MTFS-I) to increase delivery of high-quality FBS in ASU treatment settings; the evidence base supporting each component of MTFS-1 is presented in the next sections. The protocol will experimentally compare two research-based implementation strategies for sustaining MTFS-I in clinical practice: Core Training versus Core + Facilitation.

### Measurement: leveraging pragmatic quality metrics via a therapist-report measure of family participation and family therapy technique adherence

Arguably, the first step toward enhancing FBS delivery in routine care is identifying quality metrics that can reliably track FBS fidelity. This study will employ a research-validated measure of FBS fidelity, the *Inventory of Therapy Techniques for Adolescent Behavior Problems* (ITT-ABP) [15–17], that assesses both family participation and family therapy techniques. The ITT-ABP (detailed in the “Methods/design” section) is a therapist-report tool with three significant features. First, it meets key criteria for being a pragmatic measure [18]: relevance to stakeholders, low burden, broad applicability, strong psychometrics, and useful for data-driven decision-making (i.e., actionable). Second, it measures “core elements” of FBS [19]: discrete treatment techniques that are common ingredients of multiple manualized family therapy models. Core elements are considered easier to master than full manuals, and they equip clinicians with a diverse portfolio of techniques that can be judiciously applied to clients presenting with comorbid, heterogeneous, and/or emerging clinical problems [20], making them well suited for the eclectic treatment practices that constitute usual care [21]. Third, as described below, the ITT-ABP is one of few therapist-report fidelity measures in behavioral health to demonstrate promising reliability with gold-standard observational ratings, and it has shown both construct and predictive validity in usual care.

### Training: upgrading therapist-report reliability by mimicking observational coding methods

Training clinicians to reliably self-report about treatment implementation is a pragmatic strategy to enhance fidelity monitoring and ultimately increase EBI delivery [6, 22]. Unfortunately, studies attempting to show concordance between therapist self-ratings and observer ratings of fidelity have mostly produced disappointing results, casting doubt on whether therapists can reliably rate their own performance [23]. This includes research-trained clinicians delivering manualized treatment [24, 25] as well as front-line clinicians in routine care [26, 27].

MTFS-I contains an innovative *Training* component with the goal of increasing the reliability of therapist-report fidelity tools such as the ITT-ABP. As detailed below, MTFS-I utilizes online training methods to coach community clinicians to be fluent in fidelity self-rating by employing procedures analogous to those used to train observational fidelity raters in controlled studies [28]. Gold-standard observational methods, used primarily in research settings to confirm the integrity of experimental treatments, require numerous hours for introducing the coding scheme, reviewing recordings to calibrate scoring, and convening meetings throughout coding activities to prevent coder drift [28]. Directly transporting these methods to everyday care—that is, training agency staff on site to reliably assess treatment delivery by their supervisees or colleagues—is beyond the normal resource capacity of most providers. However, some have asserted that by mimicking observational methods, it may be possible to improve the reliability (and hence, utility) of therapist self-reports [6, 23, 27]. The approach most likely to succeed is online training, wherein reporter training content can be presented in a pragmatic, user-tailored manner [29]. MTFS-I online training procedures for therapist self-reporting on FBS delivery are described in the “Methods/design” section.

### Feedback system for implementation: adapting measurement feedback systems to serve as quality procedures for treatment implementation

To allow therapist-report data on FBS delivery to be regularly monitored for quality purposes, MTFS-I embeds therapist reports within a governing learning system adapted from conventional measurement feedback system (MFS) procedures. MFS is a performance feedback loop in which a given quality metric is continuously monitored by the clinician to gauge case progress and support clinical decision-making. MFS feedback loops usually take the form of easy-to-digest data reports providing summary appraisals of individual client progress on quality metrics in comparison to a desired benchmark [30, 31]. To date, MFS has been used in behavioral healthcare primarily to monitor client outcomes, wherein the metrics are standardized measures of client functioning—for example, therapists tracking weekly client-report depression scores compared to clinical norms on a validated depression scale. MFS has led to impressive gains in treatment outcomes across diverse clinical samples [32], including substance users (e.g., [33]). MFS successes for client outcomes have generated enthusiasm about the value of developing complementary procedures for treatment implementation. When attuned to implementation characteristics such as EBI fidelity, MFS can serve as a functional quality procedure with broad dissemination potential [22, 31].

MTFS-I expands on conventional MFS by (a) adding *Training* in therapist-report accuracy and (b) focusing on *Implementation* in the form of FBS fidelity. Note that the Training and Feedback components contained in MTFS-I are symbiotic: reporter training is meant to ensure that therapists generate valid data to anchor feedback reports, and feedback reports supply motivational context for dedicated participation in reporter training.

### Enhancing sustainability: applying Core Training and Facilitation strategies to boost MTFS-I implementation in usual care

To implement MFS successfully in behavioral healthcare settings, supportive strategies are needed: conducting on-site MFS training aimed at improving user attitudes and self-efficacy, incentivizing regular MFS use, engaging leadership and identifying local champions to bolster MFS use, and providing technical assistance [34–36]. To promote routine uptake of MTFS-I, this study will test two validated implementation strategies for sustaining quality procedures in behavioral care: Core Training versus Core + Facilitation. Core Training, which is based on principles of process improvement [37] and typically consists of workshop training followed by ongoing technical assistance, represents the primary implementation strategy used in large-scale innovation diffusion. Facilitation is an additive implementation strategy intended to reinforce core training and foster high-quality sustainability of the innovation [38]. Both strategies are innovation diffusion approaches that complement quality methods commonly employed in behavioral care [40]. Similar implementation interventions have improved service quality outcomes in large substance use treatment systems (e.g., [41]). For the current study, these strategies will be deployed to promote MTFS-I in ASU treatment sites in a manner that is both standardized and tailored to the unique context and self-defined needs of each site, thereby allowing MTFS-I to be scalable across several settings.

### Specific aims

This study protocol addresses the need to delineate practical, effective, and sustainable methods for promoting delivery of high-quality FBS in ASU treatment sites via installation of the MTFS-I learning system. Study aim 1 will compare Baseline versus Implementation phases in average FBS delivery (combining across sites). We hypothesize that FBS delivery will be significantly greater during the Implementation phase, following Core Training. Study aim 2 will experimentally test the effects of implementation strategies on FBS delivery. We hypothesize that Core + Facilitation will be superior to Core Training in uptake of MTFS-I components: submission of fidelity data, completion of online training activities, and review of feedback reports. Also, Core + Facilitation will be superior to Core Training in family member involvement in treatment and clinician utilization of family therapy techniques. Study aim 3 will experimentally test the effects of implementation strategies on client outcomes. We hypothesize that Core + Facilitation will be superior to Core Training in reducing substance use and increasing treatment goal achievement.

## Methods/design

### Study design

The study design is a two-group, parallel cluster randomized trial with baseline comparison: following a 4-month Baseline phase, ten ASU treatment sites will be randomized across two conditions, Core Training versus Core + Facilitation, for a 1-year Implementation phase. Cluster randomized trials are appropriate when interventions are allocated randomly to organizational units (in this case, treatment sites) rather than to individuals to enhance ecological validity [42]. For aims 1 and 2, we will collect FBS quality data along with MTFS-I uptake data. For aim 3, we will extract client outcome data from archived state databases for 1-year pre-Implementation (prior to Core Training activities) and 1-year follow-up. In aim 1, by comparing Baseline versus Implementation phases, we can examine the effectiveness of the Core Training strategy for enhancing FBS quality across all study sites, thereby yielding proof-of-concept data in accord with the well-established stage model of behavioral treatment development [43]. In aims 2 and 3, by experimentally comparing Core + Facilitation versus Core Training, we can test the value of additional time and resource expenditures embodied in Facilitation procedures for maximizing intervention benefits in FBS quality and client outcome, in line with established principles of facilitating system-level behavioral interventions [37].

### Study sites, sample size, and randomization procedures

**Fig. 1:**
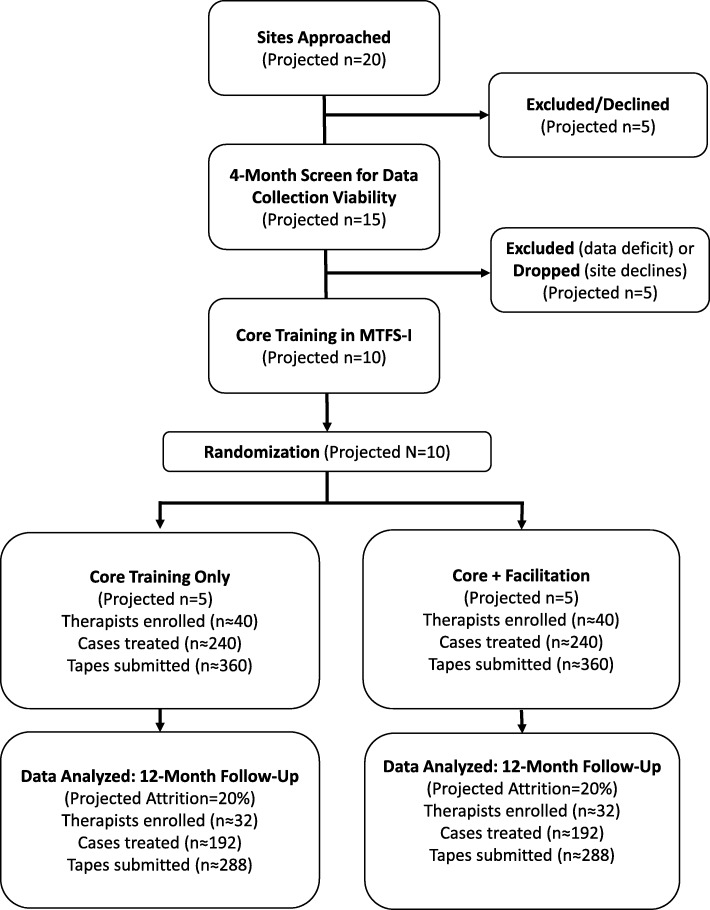
CONSORT diagram of projected study enrollment

**Fig. 2:**
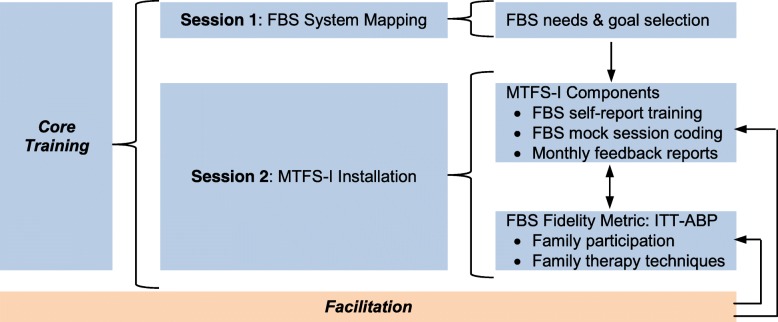
Model of proposed MTFS-I intervention components and its FBS quality metric

Ten outpatient ASU treatment sites will be purposively sampled to promote site diversity in size and location. Based on these factors, along with data on organizational characteristics of selected sites (see below), we will create yoked pairs for randomization so that each pair is matched as closely as possible (e.g., sites that are both small, rural, and with a single clinical supervisor). Randomization to Core Training or Core + Facilitation will occur after each pair has completed Core Training. State census data indicate that the median annual enrollment across substance use programs serving youth age 13–21 years is about 120. Thus for aim 3 analyses of archived state data, the study will include approximately 1200 clients across ten clinics. For aim 2, we expect to enroll 80 therapists total (8 therapists per site [6 active slots plus 2 to replace dropouts]) treating 480 total cases (60 therapist slots × 8 cases/year) for which ITT-ABP data will be provided. We anticipate that treatment will average ~ 6 sessions/case, yielding 2880 submitted fidelity checklists. For observational fidelity analyses, therapists at each site will submit audio-recorded sessions with consenting families; conservatively, about half of study families (*n* ~ 240) will consent to allow recording, and about half of sessions with completed fidelity checklists will have a companion recording, yielding a final pool of *n* ~ 721 recordings. See Fig. 1 for a CONSORT flow chart of projected study enrollment. See Fig. 2 for an outline of the components of the MTFS-I learning system, along with the two implementation strategies (Core Training, Facilitation), all of which are described in detail below.

### Study interventions: MTFS-I components as pragmatic quality procedures

#### MTFS-I online training components

Two online MTFS-I components are used to increase validity in self-reporting on FBS fidelity and also model quality of FBS delivery. Online training is a user-friendly, cost-effective way to reach large numbers of trainees and to manage continuous trainings [29]. It has proven comparable or superior to in-person workshops in increasing clinical knowledge, self-reported use of treatment skills, and clinical proficiency [44–46]. Front-line clinicians report comfort with online training, believe it to be efficacious [47], and believe it increases training accessibility and engagement [46]. Thus, online methods appear to be an excellent and practical training medium for improving the capacity of providers to recognize high-fidelity FBS and report accurately on their own FBS delivery.

MTFS-I training components are delivered weekly to therapists and supervisors. *ITT-ABP reporter training modules*: MTFS-I contains brief illustrative descriptions and related clinical exemplars describing family therapy scale items from the ITT-ABP. Each module covers 2–3 items and will conclude with learning quizzes with corrected scoring feedback. Reporter training also includes elements aimed at reducing self-report biases of various kinds, by providing continuous training in unbiased, accurate reporting [48]. *Mock FBS session coding*: 5–8 min video segments are used to model examples of ITT-ABP items, illustrating a range from moderate to high fidelity in order to support differentiated scoring. The staff will be asked to code segments directly after completing the corresponding reporter training module for those items in order to reinforce training elements. The staff will submit ITT-ABP ratings for the segment and immediately view gold standard scores, along with an explanation justifying the gold standard scoring. As discussed above, these procedures mimic well-established observational training methods and leverage immediate corrective feedback on objectively rated samples of desired performance [34]. In addition to supporting self-report reliability, these methods have strong potential for increasing FBS use via observational learning mechanisms prompted by modeling of quality FBS delivery depicted in the segments [49]. Although live coaching and guided skills practice are the most effective means to acquire new clinical skills [45], video-based modeling has shown promise for increasing use of EBIs of several kinds (e.g., [50, 51]).

#### MTFS-I feedback component

**Fig. 3:**
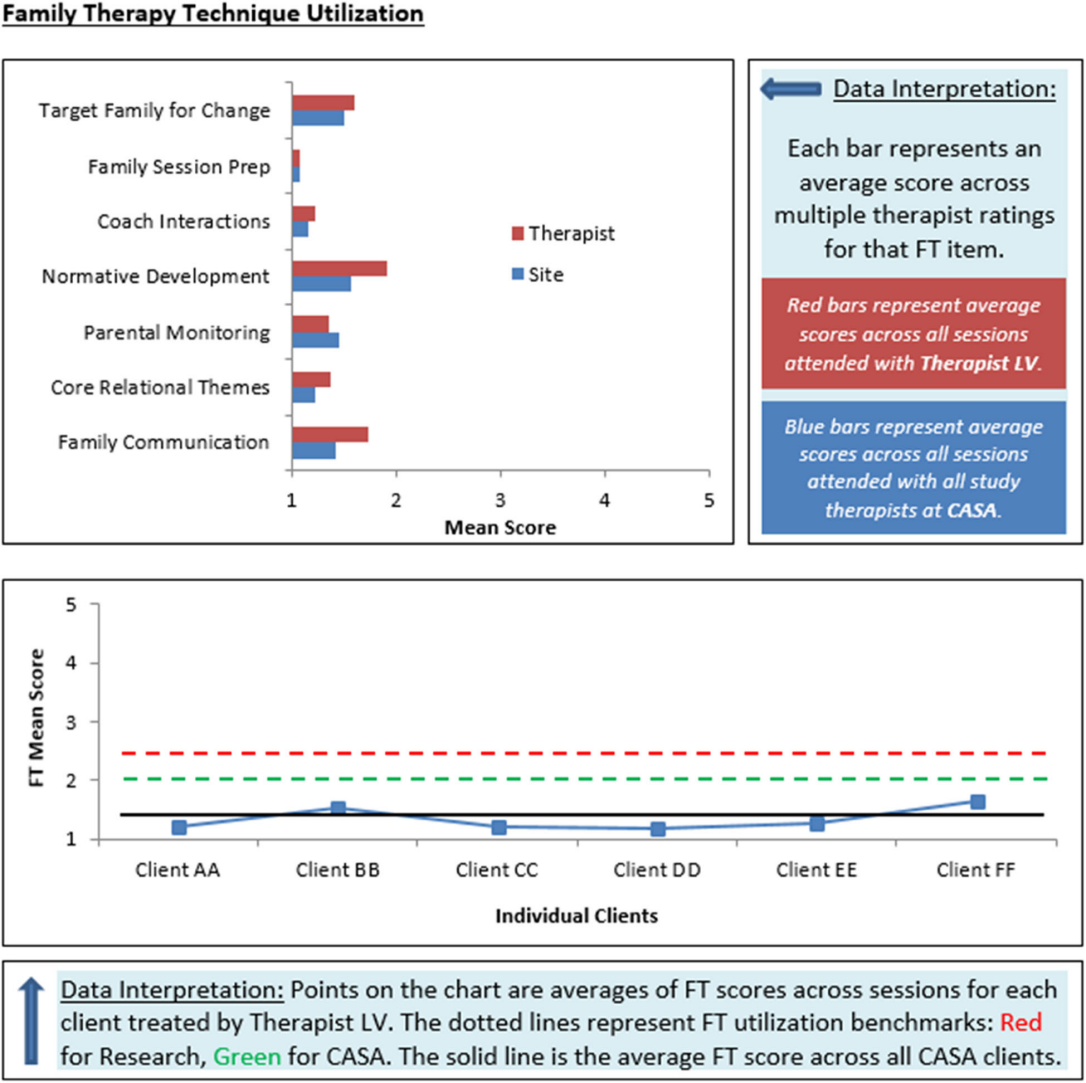
Sample of monthly feedback report based on ITT-ABP data for a site called “CASA”

MTFS-I also features *monthly feedback reports* that summarize cumulative FBS implementation for each active case, based on therapist-reported ITT-ABP data. Feedback reports contain (a) summary data on family participation in assessments, treatment sessions, family meetings, and other treatment activities; (b) mean values for each family therapy item and for the Family Therapy scale average aggregated at the client, therapist, and site levels; (c) aggregated Family Therapy scale means plotted against benchmark fidelity levels. Figure 3 depicts content from a sample feedback report. A key predictor of adoption of innovative technology is fit between the technology and service context [52]. To promote compatibility and clinical relevance and increase collaborative investment in MTFS-I, each site in the Core Training and Core + Facilitation conditions will define its own benchmark levels for FBS delivery [31] to be specified in feedback reports, along with benchmarks drawn from research studies of family therapy models [17]. These reports will spur direct comparison between therapist self-reports of FBS fidelity versus agency-specified benchmarks, precipitating data-driven self-correction responses that motivate movement toward fidelity goals [53]. Data-based case feedback along multiple dimensions, including fidelity, is also thought to optimize change potential [31]. Each site will also confer on the optimal design of feedback reports, the pragmatics of routine MTFS-I use and user-friendly depictions of quality data [6, 17], and potential organizational and staff-related facilitators and barriers to report use [35]. Feedback reports delivered to therapists will contain therapist-level data, whereas supervisor reports will contain data aggregated at the site/agency level, preserving therapist autonomy about sharing their own data in supervision.

Table 1 summarizes the user-centered technology design innovations and corresponding change mechanisms of MTFS-I components. We expect MTFS-I procedures will foster improvements in FBS fidelity for several reasons. MTFS-I will expose therapists and supervisors to high-quality FBS delivery during practice session coding, increase therapist attention to their own FBS practices and fidelity levels, allow therapists to compare self-reported fidelity levels to site-aggregated levels, and increase supervisor attention to therapist-reported FBS levels and patterns of FBS delivery over time.

**Table 1:** Technology design innovations to optimize the potency of MTFS-I components

MTFS-I component	Componen5t design innovations	Related potency boost
ITT-ABP didactic training modules	Agency selection of fidelity benchmarks for local FBS	Collaborative agency buy-in; local clinical relevance
Mock FBS session coding	Frequent video review and coding of mid-to-high quality FBS delivery	Immediate corrective feedback for coding; observational learning
Monthly feedback reports	Self-comparison to locally relevant FBS benchmarks	Cognitive dissonance re agency standards; multiple quality indices

### Study interventions: Core Training and Facilitation as implementation strategies

The study will compare two additive implementation strategies for installing MTFS-I in ASU clinics, both derived from JJ-TRIALS [38, 39]: Core Training and Core Training + Facilitation. For these strategies, data-driven decision-making is the common thread: a process by which key stakeholders in a system collect and interpret various types of data to inform decisions that will help improve or reform targeted outcomes or practices. Data-driven decision-making has been shown to increase performance and productivity across a range of industry, education, and service settings [54]. In this study, ASU sites will be trained to utilize MTFS-I as a learning management system for engaging in data-driven decision-making aimed at increasing FBS delivery and quality.

#### Core Training

We will adapt Core Training as a blended strategy toolkit [39] wherein multiple interventions targeting multiple agency roles are combined to help sites increase FBS delivery. Core Training will convene administrators, supervisors, data managers, and clinical staff for two 3-h workshop sessions. *FBS System Mapping* will focus on process mapping of existing site policies and practices for engaging family members in treatment, with the goals of completing a needs assessment to identify addressable shortcomings in current FBS and identifying FBS performance indicators already captured in site records. Mapping information will be used to collaboratively select 2–3 data-based goals for FBS quality improvement that will become exemplars during the MTFS-I Installation session. *MTFS-I Installation* will consult on integrating MTFS-I procedures into existing site procedures and data systems. It will feature principles of data-driven decision-making whereby feedback reports on FBS performance levels can be used to address FBS improvement goals selected during system mapping. Per standard practice, sites receiving Core Training will be provided routine technical assistance [7] after training is completed.

#### Facilitation

We will provide facilitation procedures in the Core + Facilitation condition for 1 year after Core Training activities. Facilitation will reinforce Core Training instruction and foster sustainability of FBS improvements over time [38]. Facilitation will feature bi-weekly phone consultation with clinical staff during which MTFS-I components (mock videos, feedback reports) are reviewed and their applicability to active cases discussed. We will also convene quarterly on-site meetings with staff serving as a local change team [37] to review progress toward FBS improvements, discuss MTFS-I feedback reports and online resource use, and provide intensive technical assistance for progressing toward site-selected FBS improvement goals. Although the Facilitation strategy requires additional resource commitments, it is a commonly used strategy shown to be feasible and valuable for scaling behavioral innovations across a variety of behavioral care systems [7, 37].

### Study measures

The ITT-ABP collects post-session therapist-report data on family participation in treatment activities (clinical assessments, treatment planning meetings, therapy sessions) and therapist use of core family therapy techniques [19]: family engagement and goal setting, relational reframing, family skills building, and coaching family interactions. It requires 2–3 min to complete and has demonstrated construct validity [15], concordance with observational ratings [16], benchmark validity for community therapists [17], and predictive validity for multiple client outcomes [55]. Client outcome data will be extracted from a state-regulated management information system used to track all client episodes of care in licensed substance use treatment programs with regard to achievement of therapeutic goals and reduction of substance use. Baseline covariates will be assessed via three therapist-report measures. *Organizational Social Context* measure [56] yields scaled scores that can be compared to national norms describing the organizational context of behavioral health clinics with regard to Organizational Culture, Organizational Climate, and Work Attitudes [57]. *Evidence-Based Practice Attitude Scale* [58] is a 15-item measure of clinician attitudes regarding the appeal of EBIs, required use of EBIs, openness to trying EBIs, and unfavorable attitudes toward EBIs [58, 59]. *Therapist Self-Reported EBI Proficiency* [15] averages therapists’ own judgments about their degree of allegiance to, and their perceived technical skill in, multiple EBIs including family therapy.

### Study procedures

#### Observational evaluation of FBS delivery

For all consenting families treated by study therapists, we will collect session audio-recordings, a minimally intrusive procedure widely accepted by families and therapists in our previous studies [26] that has proven feasible in usual care for youth behavioral health [6, 27]. From among all collected recordings, we will randomly select one session from the early phase (sessions 1–3) and later phase (sessions 4+) of each case for coding with the observer version of the ITT-ABP [16]. We will use urn randomization to balance the coding sample on study condition and site factors (census, location). With 240 clients yielding on average 1.5 recordings apiece, we anticipate coding ~ 360 sessions, of which 20% will be double-coded to establish observer reliability. If observer fidelity verification reveals that some portion of study therapists exhibit unacceptably poor self-report reliability, the observational ratings can be used to calibrate or substitute for therapist-report ratings.

#### Multireporter evaluation of implementation strategies

To verify the fidelity of the implementation strategies, Core Training and Facilitation, we will employ the JJ-TRIALS fidelity toolkit [38, 39] to evaluate implementation adherence and differentiation throughout the study. Toolkit materials include trainer- and participant-report tools assessing intervention preparation, workshop delivery of goal selection and data-driven decision-making content, knowledge and skills gained during core training, and perceived facilitation effectiveness.

#### FBS fidelity, MTFS-I uptake, and outcome data collection

ITT-ABP checklists and session recordings will be collected at all sites during the Baseline and Implementation phases. During the Implementation Phase, therapists and supervisors will be asked to confirm review of monthly MTFS-I feedback reports (distributed via email) and complete online reporter training modules and mock FBS session coding; also, therapists will report on the extent to which mock segments and feedback reports are discussed during routine clinical and peer supervision. To collect outcome data, we will use existing arrangements with the state regulatory agency for extracting archived data for all admissions of clients age 13–21 for 1 year prior to initiating Core Training activities (Baseline) and 1 year after (follow-up).

### Power analysis

With cluster trials, power is substantially affected by the number of sites and clusters (i.e., therapists) within site, and minimally by clients within cluster [42]. For aim 2 (therapist-report fidelity data), in which the unit of analysis is ITT-ABP checklists (*n* = 2880), using an effect size of 0.50 for paired contrasts between conditions, with 5 sites per comparison and 48 clients within each site, and with moderately sized intraclass correlations at levels 2 and 3 (*ρ* = 0.05) [60], yielded power of approximately 0.80*.* Our recent fidelity study [17] found the mean Family Therapy fidelity score for community therapists (2.4; SD = 0.63) was significantly higher than that for research therapists (2.0/.35). Using the standard deviation of Family Therapy fidelity scores produced by community therapists (a sample similar to the current study) and an effect size of 0.45 yields a product of 0.28, a threshold indicating meaningful change in fidelity (as illustrated by the difference found between community versus research therapist scores) that would yield a significant effect with power = 0.80. For aim 3 (archived outcome data), in which the unit of analysis is the client, for 1200 clients and assuming 10 sites and 80 therapists, an effect size of *δ* = 0.50 in line with that found similar implementation studies (e.g., [61]) yielded power = 0.80.

### Data analysis plan

Study data will have a three-level nested structure: clients within therapists, within sites (we will average across sessions for each client). The analytic approach for these nested data will be multilevel mixed-effects models examining the effects of Condition and Phase on dependent variables aggregated across each time period: 4-month Baseline phase (prior to Core Training) and 12-month Implementation phase. We will use maximum likelihood estimation for continuous variables and robust weighted least squares for categorical variables. We will model Site and Therapist as random effects in all models. We will include Site (census, location, organizational context), Therapist (age, sex, ethnicity, experience, EBI attitudes, family therapy proficiency), and Client (age, sex, race/ethnicity, family composition) factors at their respective levels to examine potential covariance effects. Variables representing adherence to Core Training and adherence to Facilitation will be entered at the Site level to ensure that fidelity differences do not confound experimental comparisons; these factors will also be tested as potential moderator variables. Effect sizes will be calculated using the standardized *d* effect size indicator, interpreted as the standardized difference between contrasts: between Conditions (Core Training vs. Core + Facilitation) and Phase (Baseline vs. Implementation) [42].

Aim 1 exploratory contrasts will examine the Phase (within-subjects) effect, contrasting Baseline versus Implementation across all sites in average FBS delivery, as measured by family participation, and family therapy technique use. Aim 2 analyses will experimentally test the effects of implementation strategies (Core Training versus Core + Facilitation) on FBS delivery. Reliability between therapists and observers on the Family Therapy technique scale of the ITT-ABP will be calculated using intraclass correlation coefficient_2,2_ [62]; we expect adequate therapist reliability (coefficients < 0.40) per our previous findings [16]. We will compare study conditions on MTFS-I uptake, family participation, and family therapy technique use. Aim 3 analyses will compare conditions on archived client outcomes for the Baseline versus Implementation phases, adjusting alpha for multiple comparisons. Product interaction terms will be included to examine Phase by Condition effects; for significant interactions, we will test simple effects by examining Phase effects separately within condition.

## Discussion

### Key study innovations

This study has several innovations that advance the science of ASU services research. MTFS-I is the first learning quality improvement system created to monitor non-manualized delivery of family therapy for ASU. MTFS-I leverages user-centered online training methods designed to strengthen the fidelity of FBS and also increase the accuracy with which therapists report on their own implementation of FBS. By increasing the capacity of therapists to report on their own FBS delivery, MTFS-I can significantly enhance the practicality and utility of methods for monitoring clinical service quality.

This study operationalizes FBS fidelity as a quality indicator for ASU treatment. Within the evolving behavioral healthcare system, quality indicators are the primary barometers used to assess the appropriateness and potential effectiveness of care [2]. Conventional quality indicators capture broad principles of care such as treatment selection, retention and follow-up rates, referrals for ancillary care, and client safety [5]. Quality indicators of this kind are important but limited: They can verify only if a given procedure occurred or a service quota was met, and not the degree of treatment fidelity delivered. In addition to tracking family participation in services, the current study also measures session-level extensiveness of treatment techniques as a quality indicator. This movement toward dimensional measurement of service quality in routine care is a top priority for improving quality procedures. For example, dimensional assessment of ASU quality affords greater specification of therapeutic processes; this, in turn, facilitates clinically rich decision-making tailored each client’s unique needs [63]. Moreover, this study employs observational methods to verify therapist self-reports of FBS technique use. Observational assessment of treatment practices is roundly needed to support data-driven upgrades to usual care [6, 22].

By focusing on core practice elements of the FBS approach, this study diverges markedly from conventional efforts to disseminate FBS for ASU via manualized family therapy models relying on purveyor-driven quality procedures. The manual-driven strategy has encountered barriers to implementing FBS models in routine care: high consultation costs, limited flexibility for selective treatment planning favored by clinicians, and sustainability limitations due to vicissitudes in local regulatory practices, purveyor commitment, and provider stamina to honor quality procedures [6, 27]. As previously discussed, the core elements approach can mitigate many of these barriers, and so far it has accumulated an impressive research base in comparison to disorder-specific treatment manuals and usual care for youth mental health problems [64, 65].

That being said, it is critical to note that MTFS-I is not intended to train therapists to implement new EBIs, nor to replace standard clinical training experiences for EBIs already in practice. As described above, MTFS-I focuses on training and feedback for EBIs that are already endorsed by, and perhaps practiced to some degree by, the given site. The goal is to promote more extensive and high-quality use of site-endorsed EBIs. MTFS-I is not designed to introduce frontline clinicians to new treatment approaches or core techniques.

### MTFS-I sustainability

Sustainability of innovations in behavioral treatment is an abiding concern of implementation science. MTFS-I procedures are sustainable beyond a research context only if (a) demands on provider time and resources are modest and (b) providers independently value its benefits and are motivated to utilize it. Regarding provider burden, MTFS-I is anchored by user-centered design features that minimize staff time commitments. The weekly time investment is 20 min for clinical staff for online training, plus another 15 min per week for therapists to enter ITT-ABP data and review feedback reports. These commitments appear feasible given the expected benefits of increased FBS quality. The flexibility of MTFS-I components also promotes their acceptability: Feedback report templates can be tailored to meet the needs of therapists, supervisors, administrators, and/or state agencies; and supervisors have appreciable latitude for how to incorporate report data into supervision meetings. The web-based learning management platform of MTFS-I is highly conducive to dynamic adaptation over time as procedures become routinized within a given agency. Finally, the proposed study is designed to test whether well-established innovation implementation strategies—core training and facilitation—enhance the sustainability of MTFS-I. Again, the time commitment for these quality procedures (six total hours for Core Training, followed weekly phone consultations and quarterly on-site meetings) seems quite favorable given the anticipated boost to the quality and maintenance of MTFS-I implementation.

Regarding provider motivation, there are several direct benefits and strong incentives for ASU treatment clinics to sustain MTFS-I. Our own research on EBIs for ASU in routine care [15–17, 26] has shown that clinicians are motivated to submit self-report data and engage in discussions related to quality improvement if they believe these activities enhance their clinical knowledge and skillsets and are valued by supervisors and agencies. Also, this study’s collaborative implementation activities will enable us to shape MTFS-I components according to local user needs [31]. These motivational processes can heighten provider commitment to adopting MTFS-I procedures, particularly when they are sanctioned by state regulatory agencies in support of government priorities for improved treatment quality and accountability. What is more, quality procedures grounded in pragmatic quality metrics such as the ITT-ABP will likely increase in value to treatment agencies as accountability contracting (e.g., value-based purchasing [66]) becomes commonplace. Finally, MTFS-I procedures bypass two major obstacles to implement MFS in routine behavioral care [35] by (a) providing ongoing and accessible training experiences to all system users and (b) ensuring that feedback data are systematically incorporated into everyday workflow and supervision.

## Abbreviations

ASU: Adolescent substance use
EBI: Evidence-based intervention
FBS: Family-based services
ITT-ABP: Inventory of Therapy Techniques for Adolescent Behavior Problems
MFS: Measurement feedback system
MTFS-I: Measurement Training and Feedback System for Implementation
